# Anti-angiogenic Effects of Metformin, an AMPK Activator, on Human Umbilical Vein Endothelial Cells and on Granulation Tissue in Rat

**Published:** 2012

**Authors:** Hamid Soraya, Nilufar Esfahanian, Yadollah Shakiba, Mahmood Ghazi-Khansari, Behroz Nikbin, Hasan Hafezzadeh, Nasrin Maleki Dizaji, Alireza Garjani

**Affiliations:** 1 *Department of Pharmacology and Toxicology, Faculty of Pharmacy, Tabriz University of Medical Sciences, Tabriz, Iran *; 2 *Department of Immunology, School of Medicine, Tehran University of Medical Sciences, Tehran, Iran*; 3 *Department of Pharmacology, School of Medicine, Tehran University of Medical Sciences, Tehran, Iran*

**Keywords:** Angiogenesis; Endothelial cells, Metformin, Migration, Proliferation

## Abstract

**Objective(s):**

Metformin is well known for activation of AMP-activated protein kinase (AMPK). AMPK activation inhibits mammalian target of rapamycin (mTOR) as a key signaling process in cell proliferation. Recent epidemiological studies demonstrate that metformin lowers the risk for several types of cancer in diabetic patients. Concerning the critical role of angiogenesis in the incidence and progression of tumors, we investigated the effect of metformin on human umbilical vein endothelial cells migration, as well as on vascular endothelial growth factor (VEGF) expressions in the cells and also on angiogenesis in air pouch model in rats.

**Materials and Methods:**

A "wound" repair method was used to assess the cell migration (n=6). Real-time PCR was performed to quantify the mRNA expression of VEGF (n=5). In air pouch model, carrageenan was injected into the air pouches on the back of rats (n=6) and following an IV injection of carmine red dye granulomatous tissue was processed for the assessment of the dye content. An ordinary ANOVA with Student-Newman-Keuls post hoc test was used to compare groups.

**Results:**

Metformin (orally, 50mg/kg) significantly (*P*<0.01) decreased angiogenesis in granulomatous tissue by 34% in pouch-bearing rats. Metformin at concentrations of 0.5-3 mM significantly (*P*<0.001) inhibited VEGF mRNA expression and endothelial cell migration. The inhibitory effects of metformin on the endothelial cell migration were reversed partially by compound C *(P*<0.01), an inhibitor of AMPK.

**Conclusion:**

The present study reported that metformin inhibited endothelial cell migration and angiogenesis *in vitro *and *in vivo*, and the effect was partially AMPK dependent.

## Introduction

Metformin is an orally administered biguanide that is commonly prescribed for the treatment of type 2 diabetes. It has been reported that the most pharmacological effects of metformin is mediated through activation of adenosine monophosphate-activated protein kinase (AMPK) (1). AMPK is a serine/threonine kinase and acts as an energy sensor in mammalian cells. Activation of AMPK blocks energy consuming (anabolic) pathways but activates energy producing (catabolic) cellular pathways (2). Recent epidemiological studies demonstrate that metformin lowers the risk for several types of cancer in diabetic patients (3-5). Many studies have demonstrated that metformin beyond the hypoglycemic action can exhibit further effects through AMPK activation including inhibition of mammalian target of rapamycin (mTOR) as a key signaling process in cells that regulates cell cycle progression, cell growth and angiogenesis. In addition, activation of AMPK by metformin is dependent on LKB1 which is a well known tumor suppressor (1). Angiogenesis and inflammation co-exist in many pathological conditions such as rheumatoid arthritis, crohn disease and tumor progression. These processes are supposed to act synergistically to develop and maintain these pathological conditions. Analysis of angiogenesis and inflammation has demonstrated that molecules such as vascular endothelial growth factor (VEGF) are essential for leukocytes activation and formation of new blood vessels (1, 6). Angiogenesis plays a major role in cancer development and is initiated in the presence of angiogenic factors such as VEGF (7). A study by Ben Sahra *et al* (8) reported that metformin can reduce cell viability up to 50% in human prostate cancer cell lines. Further several other studies also reported the inhibitory impact of metformin on colon, breast, lung, and pancreatic cancer cell lines (8, 9).

Inhibition of angiogenesis is one of the proposed actions of metformin in suppression of tumor incidence and progression. It has been reported that metformin decreases pro-angiogenic factors in polycystic ovarian syndrom and also reduces levels of VEGF in obese diabetic patients. The effects are possibly mediated through inhibition of mTOR signaling (1). Surprisingly, it has been reported that metformin in MB-435 breast cancer cell line induces angiogenesis (10). Although a number of studies have reported that metformin inhibit angiogenesis (1), future studies are needed for excluding probable pro-angiogenic effects of metformin.

Neutrophils are an important component of the immune system and have a major role in microbial infections and inflammatory responses. Several studies have reported suppressive effects of AMPK activation on inflammatory cells including macrophages and neutrophils (11-13). In spite of important role of neutrophils in eliminating microbial infection accumulation of active neutrophils, because of release of cytokines and other proinflammatory mediators, can cause tissue damage and precipitate organ dysfunction (6, 11). It has been reported that AMPK is present in neutrophils and neutrophils have important role in acute inflammatory process (11), but there is little information about the role of AMPK in acute inflammatory processes. Concerning the serious effect of neutrophils on acute inflammatory processes and also critical role of angiogenesis in the incidence and progression of tumors, we investigated the effect of metformin on neutrophil recruitment as well as on angiogenesis in carrageenen induced air pouch model in rat and VEGF mRNA expression in human umbilical vein endothelial cells (HUVECs).

## Materials and Methods

***Animals***

Wistar male rats (220-250 g, Razi, Iran) were divided into 5 groups, six in each group. Rats were housed at constant temperature (20±1.8˚C) and relative humidity (50±10%) in standard polypropylene cages, eight per cage, under a 12L:12D schedule, and were allowed food and water freely. This study was performed in accordance with the Guide for the Care and Use of Laboratory Animals of Tabriz University of Medical Sciences, Tabriz-Iran (National Institutes of Health publication No 85-23, revised 1985).

***In vivo angiogenesis assay***

To analyze the action of metformin on *in vivo* angiogenesis, the air pouch model described by Ghosh *et al* (2000) was used. Briefly rats (n=6) were lightly anesthetized with diethyl ether, the back of the rats was shaved and swabbed with 70% ethanol, and 8 ml of sterile air was injected into the subcutaneous tissue of the back in the region of the clavicles to make an oval-shaped air pouch. Twenty-four hr later, 4 ml of a 1% (w/v) solution of carrageenan (Sigma Chemical Co.) in saline was injected into the air pouch under light diethyl ether anesthesia. The carrageenan solution had been sterilized by autoclaving at 121°C for 15 min and supplemented with antibiotics [0.1 mg of penicillin G potassium (Jaber Ebn-e-Hayyan, Iran) and streptomycin sulfate (Jaber Ebn-e-Hayyan, Iran) 0.1 mg/ml of the solution] after cooling to 40-45°C. The angiogenesis was evaluated six days after carrageenan injection. Metformin was dissolved in saline and was given orally to rats at doses 25, 50, and 100 mg/kg/day 1 day before and 6 days after the carrageenan injections.

***Determination of angiogenesis in granulation tissue***

Animals were anaesthetized by IP injection of ketamin, xylazin, and acepromazin mixture, and 3 ml of 5% (w/v) carmine dye in 5% (w/v) gelatin in saline at 37°C was injected into the jugular vein of each rat, and the carcasses were chilled on ice for 3 hr. After 3 hr the entire granulation tissue was dissected, weighed, and washed with PBS (pH 7.4). The content of carmine dye in the granulation tissue as an indicator of angiogenesis was measured according to the methods described by Ghosh *et al* (14) with slight modifications. Briefly, the whole granulation tissue was homogenized in 2 volumes of 0.5 mM sodium hydroxide using a T 25 basic homogenizer (IKA Labortechnik; Italy) for 4 min at 9500 rpm on an ice bed. The tissue homogenate was centrifuged at 5,000 rpm and 4°C for 30 min; 500 µl of the supernatant was diluted 2-fold with 0.5 mM sodium hydroxide and centrifuged again at 9,000 rpm. The dye content in 200 µl of the supernatant was determined spectrophotometrically by measuring absorbance at 490 nm. For the standard curve, known amounts of carmine dye were added to the final supernatant of granulation tissue of control rats that were injected with 3 ml of a 10% (w/v) gelatin solution in saline without carmine dye, and the absorbance determined. The amount of carmine dye in the whole granulation tissue was then calculated. For visualization of granulation tissues, the tissues were fixed in 10% (v/v) formalin in PBS for 48 hr at 4°C. The samples were dehydrated by continuous immersion in 70% (v/v) ethanol for 48 hr, 90% (v/v) ethanol for 48 hr, and pure ethanol for 48 hr. After dehydration, the samples were cleared by their immersion in the cedarwood oil (Sigma Chemical Company) for 14 days. Retention of carmine dye within the vascular bed was observed with a light microscope (40× magnification).

***Determination of total leukocyte infiltration and neutrophil percentage in the pouch exudates***

In the other set of experiments (n=6) on the sixth day, the pouches were flushed with 2 ml of PBS, (pH=7.4) and vigorously massaged for 30 sec. The rats were euthanized and pouches were opened with a small incision and the exudates were collected. The total leukocyte count was determined in a Neubauer chamber and the differential cell count was determined by microscopic counting of Giemsa stained slides.

***In vitro*** **angiogenesis assay**

***Cell culture***

HUVECs were cultured in DMEM medium supplemented with 10% FBS. Culture condition was 37ºC with 5% CO_2_. When the cells reached 80% confluence, they were detached using 0.25% trypsin-EDTA and again subcultured. 

***Endothelial cell migration assay***

HUVECs were cultured in a 6-well culture plate. When the cells achieved 80–90% confluence, a wound was made on the cell area by a sterile yellow tip. Variation in the wound width within experiments was approximately 5%. After being washed with PBS, the cells were treated with a medium containing different concentrations of metformin and 2% FBS bovine serum (2% FBS allows cell survival but not cell proliferation). After 72 hr incubation, cells were washed twice with PBS, fixed by methanol and stained with Giemsa. Cell migration into the scratched area was photographed at a magnification of ×40 and quantified by calculating the difference in the denuded area using a computerized planimetry package (Landcalc, UK). Data are expressed as a percentage of the migration in untreated endothelial cells.

***RNA isolation and real-time quantitative PCR***

The total cellular RNA was extracted from the cultured cells (~1×10^5^) using Trizol. The cells were lysed in 1 ml Trizol and incubated at room temperature for 5 min. Then, 200 ml chloroform was added into the lysate, incubated for 3 min, and centrifuged for 15 min at 12,000 g at 4°C. The aqueous layer was removed, mixed with an equal volume of isopropanol and incubated for 1 hr at 4°C. The purified RNA precipitated by centrifugation at 12,000 g for 15 min and finally dissolved in 50 µl diethylpyrocarbonate (DEPC) treated water. One µg of the total RNA was converted to cDNA using the Quantitect reverse transcription kit (Qiagen- USA). Real-time PCR was performed by the Quantifast probe PCR+Rox vial kit (Qiagen- USA) using the ABI Step one plus Detection system (Applied Biosystem, USA). The cycling conditions were 45 cycles in two steps. An initial denaturation step at 95°C for 3 min, was followed by denaturation at 95°C for 3 sec, and annealing- extension at 60°C for 30 sec. For quantification, the target gene was normalized to the internal standard gene 18S. The primers were designed for detection of the VEGF-A gene expression, as given below: 

For VEGF-A, 

forward: 5'-CTTGCCTTGCTGCTCTACC-3';

reverse: 5'-CACACAGGATGGCTTGAAG-3'.

For 18S rRNA,

forward:5'- GGCTACCACATCCAAGGAA-3';

reverse: 5'- GCTGGAATTACCGCGGCT- 3'.

***Statistics***

Data were presented as mean±SD. One way ANOVA was used to make comparisons between the groups. If the ANOVA analysis indicated significant differences, a Student-Newman-Keuls post test was performed to compare the mean values between the treatment groups and the control. Differences between groups were considered significant when the *P*-values were *P*<0.05.

## Results

***Effects of metformin on angiogenesis in granulation tissue***

**Figure 1:**
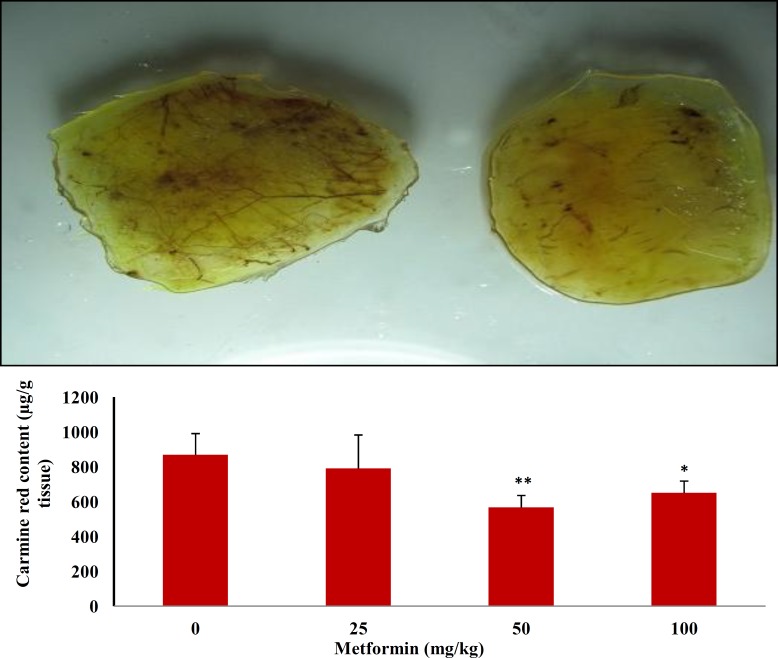
Upper trace: Effects of metformin (50 mg/kg, right) on angiogenesis in granulation tissue versus positive control (carrageenan; left) in the air pouch model of angiogenesis in rats. Lower trace: The effect of oral administration of metformin on carmine dye content (as an index of angiogenesis) in granulation tissue in the air pouch model of angiogenesis in rats. Data represented as mean SD. N=6. *P<0.05 and **P <0.01 *vs* control group (0 mg/kg metformin)

Six days after injection of carrageenan into the air pouch, a dissectible granulation tissue was formed in the subcutaneous tissue. Following intravenous injection of carmine red dye to anaesthetized animals, the dye was accumulated in the granulation tissue and the amount of the dye was assessed as an index of angiogenesis. As shown in Figure 1, oral administration of 50 and 100 mg/kg of metformin produced a significant (*P*<0.01; *P*<0.05) reduction in angiogenesis by 34 and 25%, respectively. In agreement with these findings, vascular network formation was also inhibited by metformin as shown in Figure 1 (upper trace; right). There was a greater growth of new blood vessels in the carrageenan-treated group (upper trace; left) than in the treated rats.

The effect of oral administration of metformin one day before and every day after carrageenan injection into the pouches for 6 days on the leukocytes recruitment into the exudate is shown in Table 1. Metformin had no significant effect on the total leukocyte number in the pouch exudate. However, oral administration of 25, 50, and 100 mg/kg of metformin produced a marked (*P*<0.001; *P*<0.01) reduction in the neutrophil percentage in the exudates by 13%, 18%, and 11% respectively. In the meantime, metformin also significantly (*P*<0.01) increased the lymphocyte accumulation in the pouch exudates.

***Effects of metformin on endothelial cell migration***

**Figure 2:**
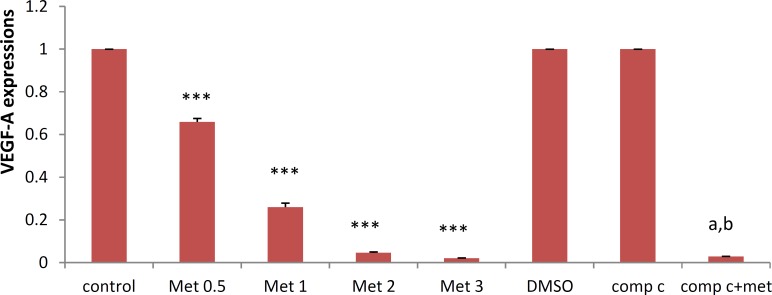
Metformin inhibits VEGF-A mRNA expressin. HUVECs were starved in serum free medium overnight before metformin treatment. Cells were then incubated with metformin (0, 0.5, 1, 2, and 3 mM) or with metformin (3 mM) in the presence of compound C (10 µM) for 72 hr. RNA was isolated and converted to cDNA. The expression of VEGF mRNA was analyzed by real time PCR. Data are mean±SD of five independent experiments.*P<0.05, **P<0.001 and *** P<0.001 *vs* control group (0 mg/kg metformin) and ^a^* P*<0.001 *vs* DMSO (control); ^b^*P*<0.001 *vs* metformin 3 mM

"Wound" repair model of migration was used to evaluate the antiangiogenic effect of metformin on endothelial cell. Confluent scrape-wounded HUVEC monolayers were incubated for 72 hr with metformin in the presence or absence of compound C, and the degree of closure of the "wound" was assessed. Metformin at concentrations of 0.5–3.0 mM induced a considerable (*P*<0.001) and concentration-dependant antiangiogenic effect indicated as inhibition of "wound" repair from 31% to 80% (Figure 2). Compound C significantly inhibited the migration (*P*<0.001), but compared with the metformin-alone-treated cells (3 mM) it partially but significantly (*P*<0.001) reversed the anti-migration effect of metformin.

***Effects of metformin on VEGF-A expressions in HUVEC***

HUVECs were incubated in different concentrations of metformin for 72 hr and the mRNA expression of VEGF-A was examined. As seen in Figure 2, metformin significantly (*P*<0.001) decreased VEGF-A mRNA levels in a concentration-dependent manner. The most marked decline in the mRNA expression was seen by 3 mM of metformin. DMSO, as a vehicle, or compound C, as an AMPK inhibitor, had no effect on the mRNA expression of VEGF however, compound C significantly (*P*<0.05) but not completely reversed the suppressive effect of metformin (3 mM) on the mRNA expression of VEGF-A in HUVECs (Figure 2).

**Table 1:** Effect of metformin (met) on leukocytes recruitment into the pouch exudates

	Control	met (25 mg)	met (50 mg)	met (100 mg)
Neutrophil percentage	84±1	73±1.9**	69±2.8**	75±1.7**
Lymphocyte percentage	16±1.5	26±1.7**	29±2.3**	22±2.2**
Total leukocyte (×10^5^)	481±71	440±109	457±118	523±41

Data represented as meanSD. N=6. *P<0.01 and **P<0.001 *vs* control group (0 mg/kg metformin)

## Discussion

Angiogenesis plays a crucial role in tumor growth and metastasis, and has been concerned as a target for intervention in cancer therapy. Metformin, an AMPK activator and antidiabetic medication, beyond its effects on glucose metabolism possess other actions, including anti-inflammatory, anti-angiogenic and anti-tumor activities (6, 15-17). Although several studies have been undertaken on the effect of metformin on the angiogenesis, especially in tumor cell lines, little parallel work has been carried out on unstimulated endothelial cells. We utilized air pouch model of carrageenin-induced inflammation as an *in vivo* model for evaluation of anti-inflammatory activities of metformin and also human umbilical vein endothelial cells (HUVEC) as an *in vitro* model for assessment of anti-angiogenic effect of metformin and the role of VEGF in this effect. In the air pouch model we demonstrated that metformin had a considerable inhibitory effect on neutrophil recruitment into the pouch exudation. Neutrophils have a major role in the inflammatory process. Neutrophils appear to be able to secrete different forms of pro-angiogenic molecules, especially VEGF-A (18). VEGF is a mitogen that has various functions on endothelial cells, including increased vascular permeability, inducing angiogenesis, endothelial cell growth, promoting cell migration, and inhibiting apoptosis (19, 20). Besides, metformin decreased red dye contents in the granulation tissue of the pouches showing a significant anti-giogenic effect. Consistent with our finding, Zhao *et al* (11) reported that the activation of AMPK decreases neutrophil proinflammatory activity. 

In the present study, i*n vitro* experiments on human umbilical vein endothelial cell migration demonstrated that metformin decreased cell migration as well as VEGF mRNA expression. 

A number of studies reported the antiangiogenic action of metformin, but most of them were done on tumor cells. Rattan *et al* (23) demonstrated that in addition to inhibiting tumor cell proliferation, metformin treatment inhibits both angiogenesis and metastatic spread of ovarian cancer. Very similar to our work is Xavier and co-workers (6) study, finding that metformin inhibited inflammatory angiogenesis in a murine sponge model by suppression of the levels of intraimplant transforming growth factor (TGF-beta1). 

By considering that metformin activates AMPK and AMPK can inhibit angiogenesis (1, 21) and neutrophil recruitment (11, 6), we hypothesized that metformin can exert anti-angiogenic effect by activation of AMPK. 

Compound C, a cell-permeable pyrazolopyrimidine derivative, acts as a potent and selective ATP-competitive inhibitor of AMPK (22). In the present study, we demonstrated that the anti-migration effects of metformin on endothelial cells as well as the inhibitory effect of metformin on the mRNA expression of VEGF-A were significantly but not completely blocked by compound C. This indicates that the AMPK pathway is involved, at least in part, in the anti-angiogenic action of metformin.

Surprisingly, compound C alone showed a slight but significant anti-proliferative and anti-migration action. These paradoxical effects in the present study probably imply the involvement of AMPK-dependent and AMPK-independent mechanisms in metformin anti-angiogenic actions. 

## Conclusions

In the light of these findings, we suggest that metformin attenuates the carragenan induced neutrophil activity and angiogenisis and has a potential effect in inhibiting the endothelial cells migration through the suppression of VEGF-A mRNA expressions. In addition, the AMPK activity, at least in part, is required for the above mentioned effects. Thus, it might be useful to target AMPK signaling in future efforts to prevent angiogenic and inflammatory disease.
